# Cecropin supplementation improves growth performance by regulating immune function, rumen fermentation and microbiota in goats

**DOI:** 10.5713/ab.25.0103

**Published:** 2025-06-10

**Authors:** Xinhong Zhou, Xiaoyun Shen

**Affiliations:** 1 School of Life Science and Engineering, Southwest University of Science and Technology, Mianyang, China; 2 Rural Revitalization Project Center, Guizhou Department of Agriculture and Rural Affairs, Guiyang, China

**Keywords:** Antimicrobial Peptide, Antioxidant Capacity, Rumen Microbiota, Serum Biochemistry, Volatile Fatty Acids

## Abstract

**Objective:**

This study aimed to determine the effects of cecropin on the growth performance, antioxidant capacity, immune function, rumen fermentation parameters, and rumen microbiota of goats.

**Methods:**

Twelve male Yudong black goats were randomly divided into two groups, with 6 replicates per group. The control group (CON) was fed a basic diet, while the antimicrobial peptide group (AMP) received a diet supplemented with 500 mg/kg cecropin. The experimental period lasted for 60 days.

**Results:**

Compared with the CON group, the AMP group showed significantly improved FW (35.46 vs. 37.33 kg, p<0.05), average daily gain (205.19 vs. 234.78 g/d, p<0.05), and reduced feed-to-gain ratio (6.45 vs. 5.66, p<0.05). The AMP group presented significantly higher SOD, GSH-Px, and CAT activities and total antioxidant capacitylevels in the serum, while the MDA content was significantly lower (p<0.05). Furthermore, compared with the CON group, the levels of IgG, IgA, and IL-10 in the AMP group were significantly increased, while the levels of IL-2, IL-6, IL-1β, and TNF-α were significantly decreased (p<0.05). In the rumen fluid, the AMP group presented significantly greater propionate and total volatile fatty acid levels, with a significantly lower acetate/propionate ratio (p<0.05). Microbial analysis revealed differences in rumen microbiota diversity and composition between the two groups. At the phylum level, the AMP group presented significantly greater abundances of *Bacteroidota*, *Fibrobacterota*, *Desulfobacterota*, and *Elusimicrobiota*, whereas the *Firmicutes* abundance was significantly lower than that in the CON group (p<0.05). At the genus level, the AMP group presented significantly greater abundances of *Prevotella*, *Rikenellaceae_RC9_gut_group*, *F082*, *Fibrobacter*, *Prevotellaceae_UCG-003*, *Bacteroidales_RF16_group*, *Christensenellaceae_R-7_group*, and *UCG-010*, whereas the abundances of *Prevotellaceae_UCG-001* and *Butyrivibrio* were significantly lower (p<0.05).

**Conclusion:**

Overall, these results suggest that adding 500 mg/kg cecropin to the diet promotes goat growth performance by improving serum antioxidant capacity and immune function, optimizing rumen fermentation parameters, and modulating rumen microbiota.

## INTRODUCTION

The 1940s was a significant period for drug development, with penicillin being the first antibiotic discovered. It effectively controls infectious diseases in clinical applications. Each year, up to 90% of global antibiotic consumption is used in food animal production and farming [1]. However, the excessive use of veterinary antibiotics can lead to antibiotic residues in animal-derived food and bacterial resistance, which poses severe public health issues. To curb the rise of “superbugs,” it is imperative to develop safe and harmless antimicrobial drugs. Antimicrobial peptides (AMPs), also known as host defense peptides, typically consist of 6 to 100 amino acids. They not only rapidly eliminate invading pathogens but also initiate the body’s immune response to further clear pathogens, playing an important role in innate immunity [2]. Compared with those of traditional antibiotics, the antimicrobial mechanisms of AMPs make it more difficult for bacteria to develop resistance. AMPs can disrupt the cell membranes of pathogens through different mechanisms. Owing to their structural characteristics and the positive charge on most of them, AMPs can interact with lipid components (hydrophobic regions) and phospholipid groups (hydrophilic regions), disrupting membrane integrity and inhibiting the activity of intracellular substances such as nucleic acids and proteins. As a result, they exhibit broad-spectrum antimicrobial activity against bacteria, fungi, viruses, and other microorganisms [3,4]. A wide variety of AMPs, such as cecropin, were discovered in 1980 and were among the first identified. To date, over 3,000 gene sequences encoding AMPs have been cataloged in AMP databases [5]. Cecropin has garnered extensive attention because of its unique antimicrobial mechanism and excellent efficacy. These peptides typically have small molecular weights and can effectively inhibit or kill various bacteria at low concentrations. The antimicrobial mechanisms of these bacteria primarily involve disrupting bacterial cell walls, inhibiting bacterial enzyme activity, and interfering with bacterial DNA synthesis [6]. Additionally, cecropin has several other advantages, including good thermal stability, no toxic side effects on host cells, and a low likelihood of developing resistance.

AMPs, as green and side effect-free antimicrobial agents, have become a new type of feed additive because of their broad-spectrum bactericidal properties and unique mechanisms of action [7]. AMPs can significantly influence livestock production performance, the gut microbiota, immune capacity, and disease prevention. Studies have shown that AMPs can be used to treat diarrhea in piglets infected with *Escherichia coli*, significantly reducing the levels of inflammatory factors IL-6 and TNF-α [8]. AMPs can improve growth performance by increasing the number of beneficial microorganisms in the cecum of piglets, regulating the mRNA expression of inflammatory factors, and enhancing gut morphology [9]. Cecropin A can effectively alleviate LPS-induced apoptosis in bovine endometrial epithelial cells by inhibiting the mitochondrial-dependent apoptosis pathway [10]. Moreover, cecropin AD can alleviate Mycoplasma pneumonia in mice induced by *Mycoplasma capricolum* subsp. *Capripneumoniae* (Mccp) by inhibiting the *TLR4-NF-κB* signaling pathway and the *Fas/FasL-caspase-8/3* signaling pathway, thus reducing lung tissue damage. To date, few studies have reported the use of AMPs as substitutes for feed antibiotics and growth promoters in ruminant nutrition. In goat farming, there has been limited research on AMPs. Therefore, this study aims to provide a theoretical basis for the application of cecropin AMPs during the fattening stage in goats.

## MATERIALS AND METHODS

### Animals, diets, and experimental design

Twelve healthy, 3-month-old male yudong black goats with similar body weights (23.19±0.32 kg) were selected and randomly divided into two groups, with 6 replicates per group, with each replicate consisting of one goat. The control group (CON group) was fed a basic diet, whereas the antimicrobial peptide group (AMP group) was fed a diet supplemented with 500 mg/kg cecropin. The diets were formulated according to the NY/T816-2021 nutritional requirements of meat sheep, with the composition and nutritional levels of the basic diet shown in Table 1. The preexperimental period lasted for 10 days, and the experimental period lasted for 60 days. Before the trial, the goats underwent shearing and deworming, and the goat housing was disinfected. The goats were housed in pens and fed twice daily at 9:00 AM and 5:00 PM, with free access to water and feed.

### Sample collection

#### Blood and rumen fluid collection

At the end of the experiment, prior to the morning feeding, 5 mL of blood was collected from the jugular vein of all goats in each group. After standing, the blood was centrifuged at 4°C and 1,300×g for 10 minutes to obtain the serum, which was stored at −20°C. Additionally, 100 mL of rumen fluid was collected via a goat oral rumen fluid collector. Of the 100 mL collected, 50 mL was immediately used to measure the rumen pH, while the remaining 50 mL was filtered through four-layer sterile gauze and then placed into sterile 50 mL EP tubes. These samples were rapidly frozen in liquid nitrogen and later stored at −80°C for future analysis.

#### Growth performance measurement

The initial body weights of the experimental goats were recorded before the trial period. On day 60 of the trial, body weight was measured prior to the morning feeding. The average daily gain (ADG) was calculated. The feed amount and leftovers were recorded daily to calculate the average daily feed intake (ADFI) and feed-to-gain ratio (F/G). The calculation formulas were as follows:


(1) ADFI (g)=Total feed intake/Number of experimental days

(2) ADG (g)=(Final body weight-Initial body weight)/Number of experimental days

(3) F/G=ADFI/ADG

#### Serum immunity and antioxidant indices measurement

Serum immunoglobulin A (IgA), immunoglobulin M (IgM), and immunoglobulin G (IgG) levels were measured via enzyme-linked immunosorbent assay (ELISA). The levels of the cytokines IL-2, IL-6, IL-10, IL-1β, and TNF-α, as well as the antioxidant markers superoxide dismutase (SOD), serum catalase (CAT), total antioxidant capacity (T-AOC), glutathione peroxidase (GSH-Px), and malondialdehyde (MDA), were measured. The kits used for these tests were purchased from Nanjing Jianchen Bioengineering Institute.

#### Rumen fermentation parameters measurement

The rumen pH was directly measured via a portable pH meter [11]. The ammonia nitrogen concentration (NH_3_-N) was determined via a colorimetric method [12]. Volatile fatty acids (VFAs) were measured by gas chromatography. Briefly, a sample was mixed with 2 mL of water (1:3 phosphoric acid solution) and vortexed for 2 minutes. Then, 2 mL of ether was added, and the mixture was extracted for 10 minutes, followed by centrifugation at 2,000×g for 20 minutes (low-temperature treatment with an ice–water bath). After centrifugation, the ether phase was collected, and 2 mL of ether was added again for extraction, followed by another 10-minute extraction and centrifugation. The ether phases from both extractions were combined and evaporated to a final volume of 4 mL for analysis.

#### Rumen microbiota 16S rDNA analysis

Genomic DNA was extracted from samples via a DNA extraction kit (Magen D6356-02), and the quality of the extracted DNA was checked via 1% agarose gel electrophoresis. For amplification of the bacterial 16S rRNA gene V3 and V4 regions, the primers 343F (TACGGRAGGCAGCAG) and 798R (AGGGTATCT AATCCT) were used. After the PCR products were obtained, amplification was initially checked via 2% agarose gel electrophoresis, and the PCR products were subsequently recovered via the AxyPrep DNA Gel Recovery Kit. The recovered products were rechecked via 2% agarose gel electrophoresis. Genome library construction and sequencing were carried out via the Illumina PE250 platform. The resulting sequence data were analyzed in depth via QIIME2 and the R language package. The alpha diversity indices of the rumen microbiota at the amplicon sequence variant (ASV) level were analyzed on the basis of the ASV table in QIIME2 and visualized in box plots. Beta diversity analysis was also performed via QIIME2. For microbiota analysis, differences in microbial relative abundances between groups were assessed using the Wilcoxon rank-sum test, and p-values were adjusted for multiple comparisons using the Benjamini-Hochberg false discovery rate (FDR) correction. Differential bacterial analysis was carried out via LEfSe software (available at: http://huttenhower.sph.harvard.edu/galaxy/).

### Statistical analyses

All data were expressed as mean±standard error of the mean (SEM). Prior to analysis, the normality of each continuous variable was assessed using the Shapiro–Wilk test, and the results indicated that the data conformed to a normal distribution. No biologically implausible outliers were detected; therefore, all data points were retained for analysis. Statistical significance was determined using independent sample t-tests in SPSS 23.0 software (IBM). Differences between treatment means were reported as significantly different at p-values*<* 0.05. To provide a more comprehensive interpretation of the results, 95% confidence intervals were calculated for key variables related to growth performance, antioxidant indices, immune markers, and rumen fermentation parameters using SPSS 23.0.

## RESULTS

### The effects of cecropin on the growth performance of goats

The effects of cecropin on the growth performance of goats are shown in Table 2. Compared with the CON group, the AMP group presented significantly greater FW and ADG values (p<0.05), and the F/G ratio was significantly lower than that of the CON group (p<0.05). There was no significant effect of adding cecropin to the diet on the ADFI (p*>*0.05).

### The effects of cecropin on antioxidant function in goat serum

The effects of cecropin on antioxidant function in goat serum are shown in Table 3. SOD, GSH-Px, and CAT activities and T-AOC levels were significantly greater in the AMP group than in the CON group (p*<*0.05), whereas the MDA content was significantly lower in the AMP group than in the CON group (p*<*0.05).

### The effects of cecropin on immunoglobulins and cytokines in goat serum

The effects of cecropin on immunoglobulins and cytokines in goat serum are shown in Table 4. The levels of IgG and IgA in the serum of goats in the AMP group were significantly greater than those in the CON group (p*<*0.05), whereas there was no significant difference in IgM levels between the two groups (p*>*0.05). The levels of IL-2, IL-6, IL-1β, and TNF-α in the AMP group were significantly lower than those in the CON group (p*<*0.05). The level of IL-10 in the AMP group was significantly greater than that in the CON group (p*<*0.05).

### The effects of cecropin on rumen fermentation parameters in goats

The effects of cecropin on rumen fermentation parameters in goats are shown in Table 5. The contents of propionate and total volatile fatty acids (TVFAs) in the rumen fluid of goats in the AMP group were significantly greater than those in the CON group, whereas the acetate/propionate ratio was significantly lower in the AMP group than in the CON group (p*<*0.05). However, adding cecropin to the diet did not have a significant effect on the pH or the NH3-N, acetate, butyrate, valerate, isobutyrate, or isovalerate contents in the rumen fluid (p*>*0.05).

### The effects of cecropin on the rumen microbiota in goats

**Figure 1:**
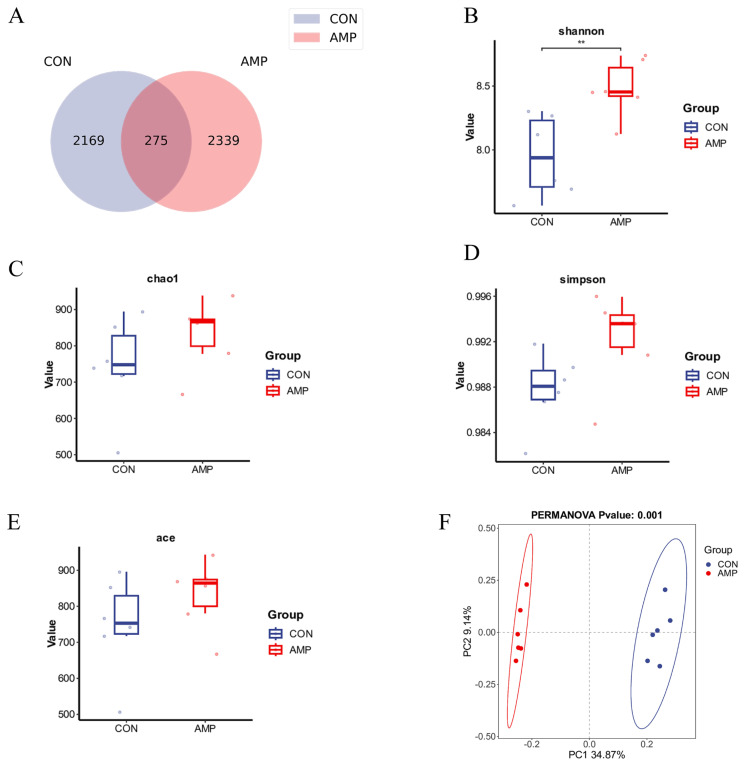
Effect of cecropin on the rumen microbiota in goats. (A) Venn diagram of the amplicon sequence variant (ASV) distribution. (B) Shannon index. (C) ACE index. (D) Chao1 index. (E) Simpson index. (F) Principal coordinate analysis (PCoA) plot. * Indicates that there is a significant difference between the two groups (* p<0.05, ** p<0.01).

We performed 16S rDNA sequencing analysis on goat rumen fluid (Figure 1). In the CON group, 2,169 unique ASVs were detected, whereas in the AMP group, 2,339 unique ASVs were detected, with 275 shared ASVs between the two groups. These findings indicate that cecropin feeding increases the number of ASVs in the goat rumen fluid (Figure 1A). Microbial alpha diversity analysis revealed that the Shannon index in the AMP group was significantly greater than that in the CON group (p<0.05, Figure 1B). Additionally, the ACE, Chao1, and Simpson indices in the AMP group also tended to increase compared with those in the CON group (p*>*0.05, Figures 1C–1E). Principal coordinate analysis (PCoA) based on the weighted UniFrac distance and beta diversity analysis revealed significant differences in the microbial community structure between the CON and AMP groups (p = 0.001). PC1 and PC2 explained 34.87% and 9.14% of the variance, respectively, and the distributions of the two groups in the plot clearly differed. These results suggest that feeding AMPs can affect the species composition of the microbial community, thereby influencing the microbial community’s function.

**Figure 2:**
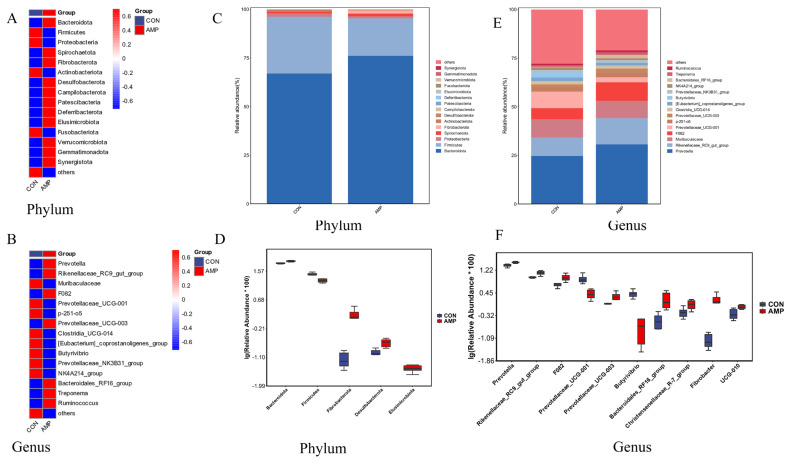
Microbial composition of goat rumen fluid and identification of differential species. (A,B) Cluster heatmap of the top 15 phyla and genera, red indicates a relatively high relative abundance of species, while blue indicates a relatively low relative abundance of species. (C,E) Microbial abundance of the top 15 phyla and genera. (D,F) Boxplot of the top 10 species with significant differences in abundance. Different colors represent different groups of samples respectively, and the ordinate represents the log-transformed values of the relative abundances of species.

We studied the differences in the microbiota at the phylum and genus levels and detected significant differences in the microbial composition between the CON and AMP groups, with distinct variations in the relative abundance of each microbial species between the two groups (Figures 2A, 2B). At the phylum level, the dominant phyla were *Bacteroidota* and *Firmicutes*, accounting for more than 95% of the total microbiota. Compared with the CON group, the AMP group presented a significant increase in the abundance of *Bacteroidota*, *Fibrobacterota*, *Desulfobacterota*, and *Elusimicrobiota*, whereas the abundance of *Firmicutes significantly* decreased (p<0.05, Figures 2C, 2D). At the genus level, the dominant genera were *Prevotella*, *Rikenellaceae_RC9_gut_group*, *Muribaculaceae*, *F082*, and *Prevotellaceae_UCG-001* (Figure 2E). We analyzed the top 10 genera with significant differences at the genus level and found that, compared with the CON group, the AMP group presented significantly greater abundances of *Prevotella*, *Rikenellaceae_RC9_gut_group*, *F082*, *Prevotellaceae_UCG-003*, *Bacteroidales_RF16_group*, *Christensenellaceae_R-7_group*, *Fibrobacter*, and *UCG-010* (p<0.05, Figure 2F), whereas the relative abundances of Prevotellaceae_*UCG-001* and *Butyrivibrio* were significantly lower (p*<*0.05, Figure 2F).

**Figure 3:**
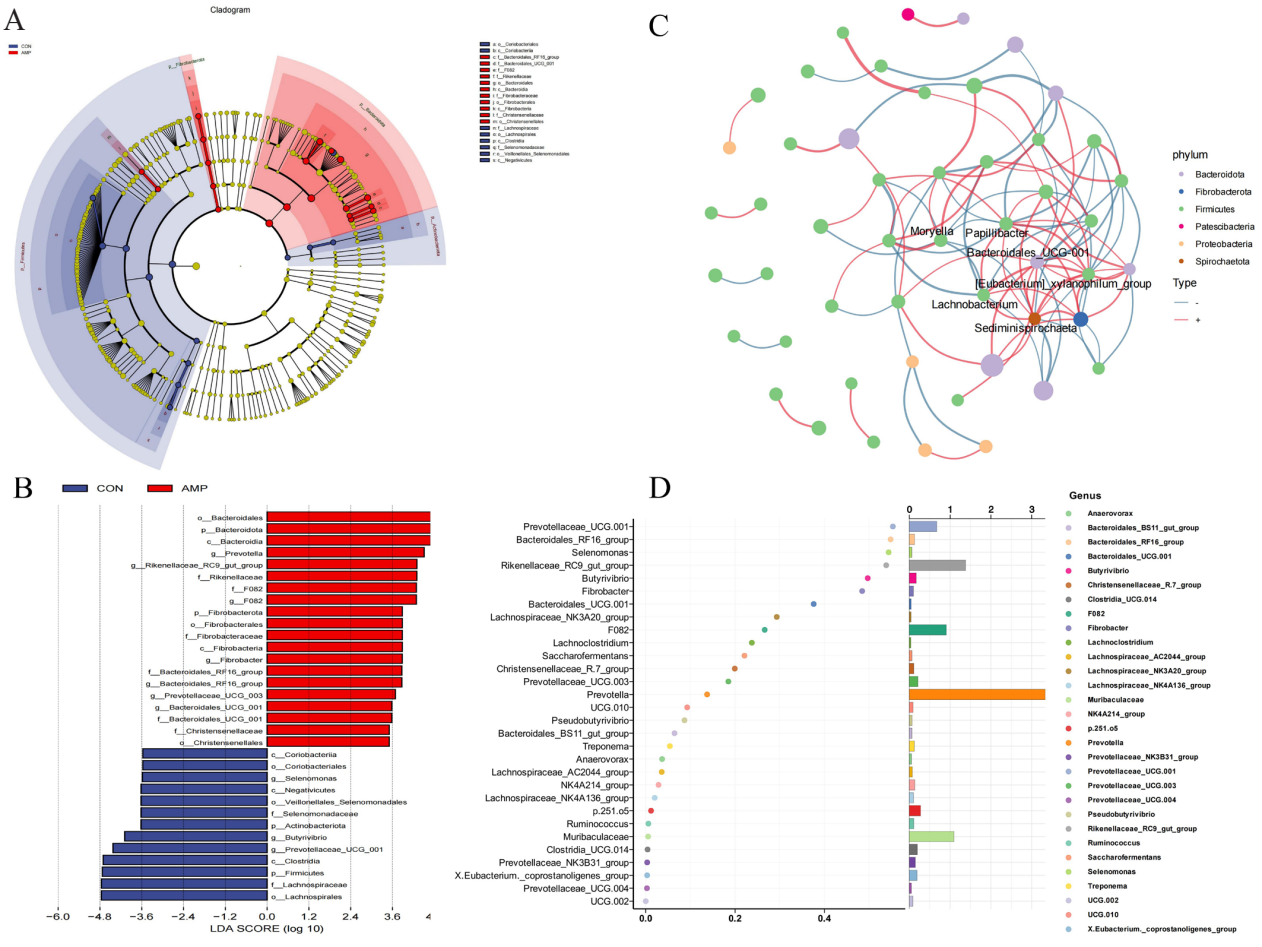
Identification of key microbes in goat rumen fluid. (A) Differential species annotation dendrogram. Different colors represent species with significantly higher abundances in different groups. The yellow nodes represent species that show no significant differences in the comparison between the two groups. The diameter of each node is proportional to the relative abundance. The nodes of each layer, from the inside out, represent phylum, class, order, family, and genus respectively. The annotations of the species markers in each layer indicate phylum, class, order, family, and genus from the outside in. (B) Differential species score plot. (C) Microbial correlation network. The size of the nodes in the figure represents the abundance of species, and different colors represent species at the phylum level. The color of the connecting lines indicates the positive or negative correlation. Red indicates a positive correlation, and blue indicates a negative correlation. The thickness of the lines represents the magnitude of the correlation coefficient. The thicker the line, the higher the correlation between species. The more lines there are, the closer the connection between the species and other species. (D) Important species dot plot based on random forest analysis, with the species (variables) importance dot plot on the left. The x-axis represents the importance measure, and the y-axis represents the species names ranked by their importance.

To analyze the effects of cecropin on specific microbial communities, we used LEfSe analysis to identify differential species between the two groups. As shown in Figures 3A, 3B, significant differences were observed in the microbial community structure between the AMP group and the CON group. The results indicated that the microbes enriched in the CON group mainly included mainly *Firmicutes*, *Lachnospiraceae*, and *Clostridia*. Additionally, the genera *Butyrivibrio* and *Selenomonas* predominated in the CON group. In contrast, the dominant microbial groups in the AMP group included *Bacteroidota*, *Fibrobacterota*, and *Rikenellaceae RC9 gut group*. Furthermore, the genera *Prevotella* and *Fibrobacter* were significantly enriched in the AMP group, suggesting that cecropin treatment may promote the growth of these fiber-degrading microbes. Overall, cecropin significantly altered the composition of the goat rumen microbial community, promoting the growth of microbes associated with fiber degradation.

Moreover, we also conducted a network analysis of the microbial community correlations (Figure 3C). The results showed that feeding cecropin significantly altered the interactions within the goat rumen microbial community, particularly by enhancing positive correlations between specific microbial groups, which promoted the synergy and functional stability of the microbial community. In this network, *Firmicutes* (green nodes) dominated, whereas *Bacteroidota* (purple nodes) and *Fibrobacterota* (blue nodes) also exhibited strong connectivity. Core genera such as *Sediminispirochaeta* and *Lachnobacterium* were located at the center of the network, potentially playing key roles in the ecological functions of the microbial community. Positive correlations were mainly observed between functionally similar microbes, whereas negative correlations were more common between microbes from different phyla, reflecting niche competition and complementarity. Additionally, via random forest analysis, we identified key microbial genera (Figure 3D). The results indicated that *Prevotellaceae_UCG.001* had the highest importance score. Other genera, including *Bacteroidales_RF16_group*, *Selenomonas*, and *Rikenellaceae_RC9_gut_group*, also presented high importance. These findings suggest that cecropin may influence the metabolic function of the rumen by modulating the abundance of specific microbial genera. These results provide a theoretical basis for further investigations of the potential benefits of cecropin on goat rumen function.

### Correlation analysis between microbiota and other indicators

**Figure 4:**
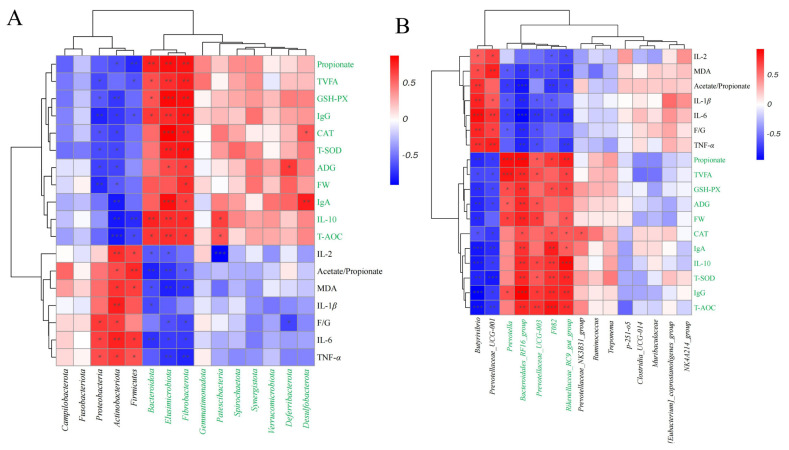
Correlation cluster heatmaps between the microbiota and other indicators. The color scale represents the strength and direction of the correlation: red indicates a positive correlation, and blue indicates a negative correlation, with darker shades representing stronger correlations. Lighter shades closer to white indicate weaker correlations. (A) Phylum-level correlation heatmap showing the relationships between the 15 most abundant phyla and other indicators. (B) Genus-level correlation heatmap showing the relationships between the 15 most abundant genera and other indicators. * Represents a correlation with p<0.05, ** represents a correlation with p<0.01, and *** represents a correlation with p<0.001. TVFA, total volatile fatty acid; GSH-Px, glutathione peroxidase; IgG, immunoglobulin G; CAT, catalase; T-SOD, total superoxide dismutase; ADG, average daily gain; FW, final body weight; IgA, immunoglobulin A; IL-10, interleukin-10; T-AOC, total antioxidant capacity; IL-2, interleukin-2; MDA, malondialdehyde; IL-1β, interleukin-1 beta; F/G, feed-to-gain ratio (g:g); IL-6, interleukin-6; TNF-α, tumor necrosis factor-alpha.

We used Spearman’s correlation coefficient to explore the associations between rumen microbiota and host physiological indicators. At the phylum level, *Fibrobacterota* exhibited significant positive associations with FW, ADG, T-SOD, GSH-PX, CAT, T-AOC, IgG, IgA, IL-10, Propionate, and TVFA (Figure 4A, p<0.05). *Elusimicrobiota* also showed significant positive associations with ADG, T-SOD, GSH-PX, CAT, T-AOC, IgG, IgA, IL-10, propionate, and TVFA (Figure 4A, p<0.05). Additionally, indicators marked in green in the figure also demonstrated positive correlations. At the genus level, *Bacteroidales_RF16_group* was significantly positively associated with FW, ADG, T-SOD, GSH-PX, CAT, T-AOC, IgG, IgA, IL-10, propionate, and TVFA (Figure 4B, p<0.05). *Rikenellaceae_RC9_gut_group* was positively associated with FW, T-SOD, GSH-PX, CAT, T-AOC, IgG, IgA, IL-10, Propionate, and TVFA (Figure 4B, p<0.05). *Prevotellaceae_UCG-003* showed positive associations with FW, ADG, T-SOD, T-AOC, IgG, IL-10, propionate, and TVFA (Figure 4B, p<0.05). These results suggest that specific microbial taxa may be involved in or respond to host physiological changes.

## DISCUSSION

An increasing number of studies have demonstrated the significant potential of AMPs as substitutes for antibiotics, especially when used as growth promoters in livestock production. The addition of 400 mg/kg cecropin AD to the diet significantly increased the ADG and ADFI of weaned piglets infected with *Escherichia coli* while simultaneously reducing the incidence of diarrhea [13]. Previous studies have indicated that the addition of AMPs significantly improved the FW and ADG of goats without changing their ADFI while also reducing the F/G. The growth-promoting effect of adding 2 g/kg was significantly superior to that of adding 3 g/kg in experimental goats [14]. Similarly, in a study where a composite AMP (recombinant porcine defensin and cecropin in a 1:1 ratio) was added to the diet of weaned Chuan Zhong black goats, it significantly improved their FW and ADG [15]. Consistent with these findings, our experiment revealed that adding cecropin significantly increased the FW and ADG of goats while reducing the F/G ratio, suggesting that cecropin promotes the growth and development of goats.

Serum antioxidant indicators reflect an animal’s physiological function and metabolic status. Free radicals can damage DNA and interfere with cell functions. SOD, a key participant in the body’s antioxidant defense, plays an essential role in the cellular antioxidant enzyme system. The higher the SOD activity is, the more effectively it is at scavenging free radicals [16]. CAT can reduce reactive oxygen species (ROS) levels by catalyzing the conversion of superoxide radicals into hydrogen peroxide, thus possessing detoxification and cell repair capabilities. GSH-Px eliminates extracellular hydrogen peroxide, participates in the transport of glutathione, and catalyzes the breakdown of lipid peroxides, producing corresponding alcohols to alleviate the damage caused by peroxides [17]. The MDA content directly reflects the extent of lipid peroxidation in the body, thereby indirectly indicating the degree of oxidative damage [18]. The results of this study revealed that Cecropin significantly increased the activity of SOD, GSH-Px, and CAT, as well as the T-AOC, while reducing the MDA content in the serum of goats. Consistent with these findings, dietary supplementation with AMPs has been shown to increase the antioxidant capacity and resistance to oxidative stress in fish [19,20], broiler chickens [21], and pigs [22], thereby supporting healthy growth. Immunoglobulins are globulins with antibody activity or chemical structures similar to those of antibodies, and they are widely present in the serum of mammals and play crucial roles in the body’s immune system. Their levels can serve as indicators for evaluating the immune function of animal serum [23]. Cytokines are divided into proinflammatory and anti-inflammatory types. The key proinflammatory cytokines include IL-1β, IL-2, IL-6, and TNF-α, which tightly regulate cell-mediated immune responses and play important roles in modulating immune functions. Additionally, IL-10, an anti-inflammatory cytokine, can effectively inhibit the overexpression of proinflammatory cytokines, thus maintaining immune homeostasis [24]. If the secretion levels of proinflammatory cytokines in the serum are too high, an excessive inflammatory response may occur, causing multiorgan damage and leading to disease. Previous studies have shown that feeding AMPs can increase the levels of IgM, IL-10, TGF-β, and SOD in the serum of weaned piglets while decreasing IL-12 levels [22]. Cecropin can reduce the protein expression of inflammatory cytokines TNF-α, IL-1β, and IL-8 in bovine endometrial epithelial cells induced by LPS, alleviating inflammation by inhibiting the MAPK pathway [10]. Moreover, the addition of AMPs to feed significantly reduces the expression levels of IL-2, IL-6, and TNF-α in the spleen of broiler chickens [25]. Consistent with previous findings in other animals, the results of this study revealed that Cecropin supplementation significantly increased the levels of IgG, IgA, and IL-10 in the serum of goats while reducing the levels of IL-2, IL-6, IL-1β, and TNF-α. These findings suggest that cecropin may enhance goat immune function and alleviate inflammation by regulating immune-related substances and inflammatory cytokine levels, which has positive implications for the health of goats.

VFAs are important fermentation products in the rumen of ruminants and are generated primarily through the fermentation of carbohydrates in the diet by rumen microorganisms. These VFAs not only provide a significant source of energy for ruminants but also act as key intermediates in various physiological processes within the body. Among VFAs, acetate, propionate, and butyrate are the main components, typically accounting for the vast majority of VFAs produced in the rumen. Acetate and butyrate are primarily involved in the synthesis of fatty acids, whereas propionate serves as a precursor for gluconeogenesis and plays a role in glucose synthesis. The ratio of acetate to propionate determines the fermentation pattern and energy utilization pathways in the rumen [26]. Our research revealed that the addition of cecropin to the diet increased the contents of propionate and TVFAs in goat rumen fluid and lowered the acetate/propionate ratio. This result is consistent with those of previous studies, which indicate that the addition of AMPs to ruminant diets can increase VFA production, improve the rumen fermentation environment, promote propionate production, and ultimately optimize rumen function, thereby improving animal growth performance [27].

The microbial community composition of ruminants is associated with their production traits. In this study, we found that Cecropin supplementation significantly increased the Shannon diversity index of goat rumen fluid. Consistent with our findings, AMPs can promote rumen health by increasing the diversity and richness of the rumen microbiota [14]. We investigated the differences in microbial abundance at the phylum level and found that after Cecropin was added, the relative abundances of *Bacteroidota*, *Fibrobacterota*, *Desulfobacterota*, and *Elusimicrobiota* significantly increased, whereas the relative abundance of *Firmicutes* significantly decreased. *Bacteroidota* can secrete various enzymes, such as glycoside hydrolases, which breakdown complex polysaccharides such as cellulose and hemicellulose in the diet into oligosaccharides and monosaccharides, providing available carbon sources and energy for the rumen microbial community [28]. *Fibrobacterota* is one of the major microbial groups responsible for cellulose degradation in the rumen, with a unique fibrous body structure on its cell surface that allows it to efficiently absorb and breakdown cellulose, converting it into cellobiose and glucose, playing a key role in the digestion of roughage in ruminants [29]. The increased relative abundances of *Desulfobacterota* and *Elusimicrobiota* can increase sulfur metabolism, affect rumen energy metabolism, enrich metabolic functions, and strengthen community stability [30,31]. On the other hand, the decreased relative abundance of Firmicutes may be beneficial for regulating fermentation acid production and altering microbial interactions [32].

Additionally, we found that feeding cecropin significantly increased the abundance of *Prevotella*, *Rikenellaceae_RC9_gut_group*, *F082*, *Prevotellaceae_UCG-003*, *Bacteroidales_RF16_group*, *Christensenellaceae_R-7_group*, *Fibrobacter*, and *UCG-010* in the goat rumen fluid, whereas the abundance of *Prevotellaceae_UCG-001* and *Butyrivibrio* significantly decreased. Consistent with previous research, AMPs can increase the abundance of *Fibrobacter* in rumen fluid, and an increase in *Fibrobacter* abundance may increase cellulose degradation [14,33]. *Prevotella* mainly participates in the degradation of hemicellulose, starch, and proteins and produces propionate and acetate. The increase in its abundance explains the increased propionate levels observed in the rumen fluid [34]. *Rikenellaceae_RC9_gut_group* and *Christensenellaceae_R-7_group* are important cellulose-degrading microbial groups, and their increased abundance may indicate enhanced digestion of coarse fibers in the rumen [35]. The increase in *Bacteroidales_RF16_group and UCG-010* may be related to fatty acid synthesis and metabolism [36]. Microorganisms like *Prevotella* and *Fibrobacter* play a dominant role in cellulose degradation, while the decrease in *Prevotellaceae_UCG-001* may be related to microbial competition and the intensity of cellulose degradation in the rumen [33]. The significant decrease in *Butyrivibrio* may impact rumen acidity and the balance of VFAs. *Butyrivibrio* is a major producer of butyrate, and its reduction may lead to a decrease in butyrate production, which corresponds with our observation of a decrease in butyrate levels in the rumen fluid, although this decrease was not statistically significant. Overall, the changes in the relative abundance of these microbes are crucial for maintaining the stability of the rumen environment, improving feed utilization, and promoting goat health. LEfSe analysis revealed that biomarkers of the AMP group were mainly cellulose-degrading bacteria, indicating that feeding with cecropin may increase the ability of goats to digest fiber in coarse feed. Core genera such as *Sediminispirochaeta* and *Lachnobacterium* were located at the center of the network, showing a high correlation with many other microbes, suggesting that these bacteria play a key role in maintaining the structural and functional stability of the rumen microbial community through interactions with other microbes [37,38].

AMPs are known to modulate the rumen microbiota and stimulate the microbiome to perform various functions, thereby improving the overall health of the host. In this study, we used Spearman correlation analysis to explore the relationships among goat growth performance, immune function, and the microbiota. The results indicated that after feeding cecropin, the relative abundances of *Fibrobacterota*, *Bacteroidales_RF16_group*, *Rikenellaceae_RC9_gut_group*, and *Prevotellaceae_UCG-003* in the rumen fluid were positively correlated with several beneficial indicators. These findings suggest that cecropin can significantly enhance goat growth performance and health by modulating the structure and function of the rumen microbiota. Specifically, *Fibrobacterota*, a key phylum for cellulose degradation, may have enhanced cellulose degradation efficiency and promoted the synthesis of VFAs (such as acetate and propionate), thus providing more energy for the host [39]. *Prevotellaceae_UCG-003* and *Bacteroidales_RF16_group* are closely related to carbohydrate metabolism, with the genus *Prevotella* optimizing energy utilization efficiency through branched-chain amino acid metabolism and propionate production, directly contributing to improved ADG [34]. Additionally, *Rikenellaceae_RC9_gut_group* may improve intestinal barrier function by regulating lipid metabolism and exerting anti-inflammatory effects, thus reducing pathogen colonization [40]. These microbial groups, through their synergistic actions, enhance the digestion and absorption of nutrients while increasing antioxidant and immune-regulating functions, thereby promoting goat growth performance [27,41]. In summary, cecropin achieves dual optimization of host growth performance and metabolic health by reshaping the functional network of the rumen microbiota, providing significant theoretical support for antibiotic replacement strategies.

In addition to the biological findings, the practical implications of cecropin supplementation for livestock production warrant consideration. Cecropin, as a type of AMP, offers a promising alternative to traditional antibiotics due to its broad-spectrum antimicrobial activity and low likelihood of inducing resistance. However, the cost-effectiveness and feasibility of large-scale application in commercial farming systems require further evaluation. Currently, the cost of synthesizing cecropins remains relatively high, which may limit their immediate adoption; advances in recombinant expression and peptide production technologies may help reduce costs in the future. Moreover, the risk of resistance development, though lower than that of conventional antibiotics, should still be monitored through long-term use studies. Regarding experimental conditions, all goats were raised under identical housing, feeding, and management environments to minimize the influence of external factors. Nevertheless, future studies across different farm settings and seasons will help validate the consistency and robustness of the observed effects.

It should be noted that the relatively small sample size (n = 6 per group) may limit the statistical power of the study, particularly in detecting subtle effects on microbial taxa and physiological parameters. While several significant differences were observed, these findings should be interpreted with caution and validated in larger-scale trials. Future studies with expanded sample sizes are warranted to confirm the reproducibility and generalizability of these results.

## CONCLUSION

**Figure 5:**
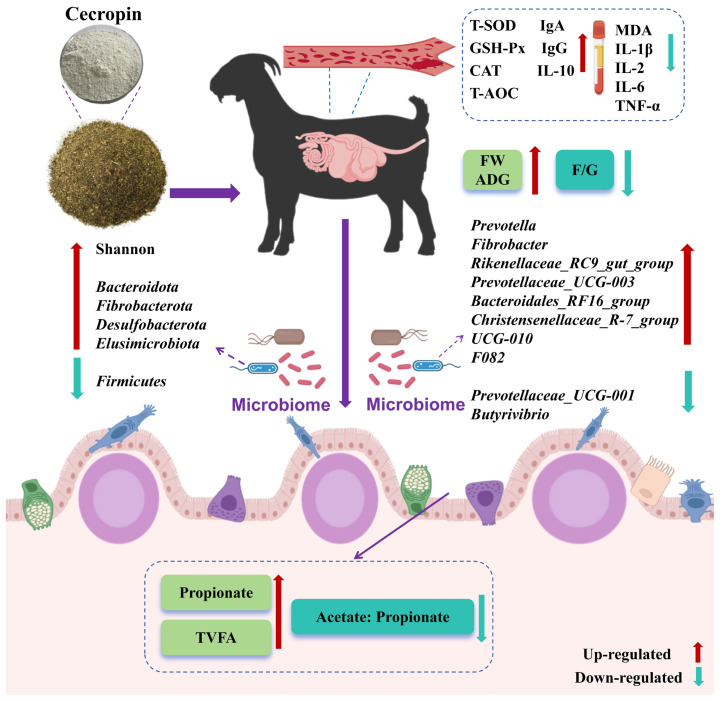
Mechanisms of the impact of dietary cecropin on the health of goats. T-SOD, total superoxide dismutase; GSH-Px, glutathione peroxidase; CAT, catalase; T-AOC, total antioxidant capacity; IgA, immunoglobulin A; IgG, immunoglobulin G; IL-10, interleukin-10; MDA, malondialdehyde; IL-1β, interleukin-1 beta; IL-2, interleukin-2; IL-6, interleukin-6; TNF-α, tumor necrosis factor-alpha; FW, final body weight; ADG, average daily gain; F/G, feed-to-gain ratio; TVFA, total volatile fatty acid.

This study demonstrates that dietary cecropin supplementation can effectively improve growth performance, antioxidant capacity, immune function, and rumen fermentation in goats (Figure 5). These effects are associated with beneficial shifts in rumen microbiota composition, suggesting that cecropin holds promise as a functional feed additive and potential antibiotic alternative to enhance goat health and productivity in sustainable livestock systems.

## Figures and Tables

**Table 1:** Composition and nutrient levels of diets (DM basis)

Ingredients	Content (%)	Nutrient levels^1)^	Content (%)
Corn silage	27.43	Crude protein	11.83
Corn stalk	7.55	Ether extract	3.25
Corn	25.10	Metabolizable energy (MJ/kg)	10.83
Wheat bran	5.18	Neutral detergent fiber	44.35
Soybean meal	9.24	Acid detergent fiber	22.41
NaHCO_3_	2.00	Calcium	0.62
Premix^2)^	2.00	Phosphorus	0.39
NaCl	1.00		
Alfalfa hay	20.50		
Total	100		

1) The metabolic energy level was a calculated value, and the others were measured values.

2) The premix provided the following per kg of the diet: VA 1, 400 IU; VD_3_ 500 IU; VE 50 IU; niacin 4.5 mg; pantothenic acid 5.25 mg; biotin 0.2 mg; Fe 27.5 mg; Cu 5 mg; Mn 125 mg; Zn 50 mg; I 0.2 mg; Se 0.16 mg.

**Table 2:** Effects of cecropin on the growth performance of goats

Items	CON^1)^	AMP^1)^	SEM	p-value
IW (kg)	23.15	23.24	0.13	0.651
FW_60_ (kg)	35.46^b^	37.33^a^	0.41	0.011
ADFI (g/d)	1,318.90	1,323.80	1.95	0.107
ADG (g/d)	205.19^b^	234.78^a^	5.55	0.004
F/G	6.45^a^	5.66^b^	0.15	0.004

1) CON, control group fed the basal diet; AMP, fed the basal diet supplemented with 500 mg/kg cecropin.

a,b Different superscript letters within a row indicate significance (n = 6, p<0.05).

SEM, standard error of the mean; IW, initial body weight; FW, final body weight; ADFI, average daily feed intake; ADG, average daily gain; F/G, feed to gain ratio.

**Table 3:** Effects of cecropin on antioxidant function in goat serum

Items	CON^1)^	AMP^1)^	SEM	p-value
SOD (U/mL)	39.48^b^	56.34^a^	2.69	0.001
GSH-Px (U/mL)	62.06^b^	75.43^a^	2.25	0.002
CAT (U/mL)	3.31^b^	7.69^a^	0.42	<0.001
T-AOC (mM)	0.84^b^	0.94^a^	0.01	<0.001
MDA (umol/L)	5.87^a^	3.16^b^	0.20	<0.001

1) CON, control group fed the basal diet; AMP, fed the basal diet supplemented with 500 mg/kg cecropin.

a,b Different superscript letters within a row indicate significance (n = 6, p<0.05).

SEM, standard error of the mean; SOD, superoxide dismutase; GSH-Px, glutathione peroxidase; CAT, catalase; T-AOC, total antioxidant capacity; MDA, malondialdehyde.

**Table 4:** Effects of cecropin on immunoglobulins and cytokines in goat serum

Items	CON^1)^	AMP^1)^	SEM	p-value
IgG (g/L)	10.32^b^	13.07^a^	0.34	<0.001
IgM (g/L)	2.12	2.03	0.10	0.559
IgA (g/L)	2.13^b^	2.67^a^	0.09	0.002
IL-2 (ng/L)	24.25^a^	19.86^b^	0.97	0.010
IL-6 (ng/L)	55.56^a^	49.11^b^	0.88	<0.001
IL-10 (ng/L)	8.97^b^	10.33^a^	0.25	0.004
IL-1β (ng/L)	97.65^a^	87.62^b^	2.04	0.006
TNF-α (ng/L)	164.15^a^	137.44^b^	2.24	<0.001

1) CON, control group fed the basal diet; AMP, fed the basal diet supplemented with 500 mg/kg cecropin.

a,b Different superscript letters within a row indicate significance (n = 6, p<0.05).

SEM, standard error of the mean; IgG, immunoglobulin G; IgM, immunoglobulin M; IgA, immunoglobulin A; IL-2, interleukin-2; IL-6, interleukin-6; IL-10, interleukin-10; IL-1β, interleukin-1β; TNF-α, tumor necrosis factor-α.

**Table 5:** Effects of cecropin on rumen fermentation parameters in goats

Items	CON^1)^	AMP^1)^	SEM	p-value
PH	6.32	6.33	0.16	0.954
NH_3_-N/(mg/dL)	22.76	20.80	0.80	0.137
Acetate/(mmoL/L)	54.56	54.60	1.23	0.981
Propionate/(mmoL/L)	24.32^b^	31.72^a^	1.31	0.003
Butyrate/(mmoL/L)	6.58	6.41	0.16	0.494
Valerate/(mmoL/L)	0.90	0.92	0.04	0.816
Isobutyrate/(mmoL/L)	6.00	5.93	0.11	0.687
Isovalerate/(mmoL/L)	1.53	1.50	0.03	0.535
Acetate/Propionate	2.27^a^	1.75^b^	0.12	0.010
TVFA	93.71^b^	101.25^a^	1.52	0.006

1) CON, control group fed the basal diet; AMP, fed the basal diet supplemented with 500 mg/kg cecropin.

a,b Different superscript letters within a row indicate significance (n = 6, p<0.05).

SEM, standard error of the mean; NH_3_-N, ammonia nitrogen; TVFA, total volatile fatty acid.
